# Our experience of anti-interleukin 1 therapy

**DOI:** 10.1186/1546-0096-13-S1-P210

**Published:** 2015-09-28

**Authors:** N Gulez, B Sozeri, P Gulez

**Affiliations:** 1 Dr. Behçet Uz Education and Research Hospital, Pediatric İmmunology, Izmir, Turkey

## Introduction

Cryopyrin-associated periodic syndromes (CAPS) are characterized by apparently unprovoked attacks of fever, rashes, and musculoskeletal and sensorineural inflammation accompanied by high acute-phase reactants. Excessive interleukin-1 (IL-1) signaling appears to be a constant feature in the pathomechanism of the disease, driven by a gain-of-function mutation in the NLRP3 gene. Familial Mediterranean fever (FMF) is a auto-inflammatory disorder characterised by recurring, self-limited episodes of fever and serositis resulting in abdominal, chest, joint and muscular pain due to mutation of MEFV. Colchicine is generally safe, nevertheless, 5-10% patients do not respond to treatment. IL-1blockade is the treatment of choice of CAPS and colchicine resistant FMF. IL-1 receptor antagonist-anakinra, human dimeric protein that incorporates the extra-cellular domain of IL-1 receptor and IL-receptor accessory protein-rilonacept, and a human anti-IL-1b monoclonal antibody-canakinumab are biologic agents used for this aim.

We report the effect of anti-interleukin 1 treatment (canakinumab, anakinra) in 12 patients (2 CAPS cases and 10 FMF cases of resistant to colchicine therapy).

## Patients and methods

We evaluated 12 anti-IL1 used patients diagnosed CAPS and FMF. Their demographic findings, the course of the disease, genetic analysis, therapy, were recorded from their hospital records.

## Results

Two patients (1 male, 1 female) were diagnosed with Systemic Onset Poliarticular Juvenile Idiopathic Arthritis when they were 2 years old. They were treated with this diagnose up to two years. After the reevaluation of patients according to the new literature, they diagnosed CAPS and canakinumab were used for therapy. Both of them has been followed for 1.5 year with clinical and laboratory remission. Ten FMF patients were evaluated for therapy in this study, and we observed that 9 of them have a homozygous M694V mutation and one of them has got M694V/M680I coumpound heterozygous mutation. Their age, symptoms onset age, and age at the diagnosis were 12.8±5, 3.2±1.9, 6.7±3.4 years respectively. The duration of colchicine was 5.9±3.5 year. We used canacinumab (5 patients) and anakinra (5 patients) because their disease was resistant to colchicine therapy. The median duration of IL1 inhibitors was 7 months. All of them has been followed with clinical and laboratory remission.

## Conclusions

Interleukin-1 inhibitors should be selected for treatment of CAPS and they may be good candidates when looking for an alternative or supplementary treatment to colchicine in FMF. These observations highlight the need for controlled trials to further evaluate the safety and efficacy of interleukin-1 antagonists in FMF patients.

